# Supplementary material for: IgG+ Extracellular Vesicles Measure Therapeutic Response in Advanced Pancreatic Cancer
Source: Cells. 2022 Sep 8;11(18):2800. doi: 10.3390/cells11182800 (PMC9496671; doi:10.3390/cells11182800)
Supplement: Supplementary file 1 [file cells-11-02800-s001.zip › cells-1832211-supplementary.pdf]

Supplementary Material

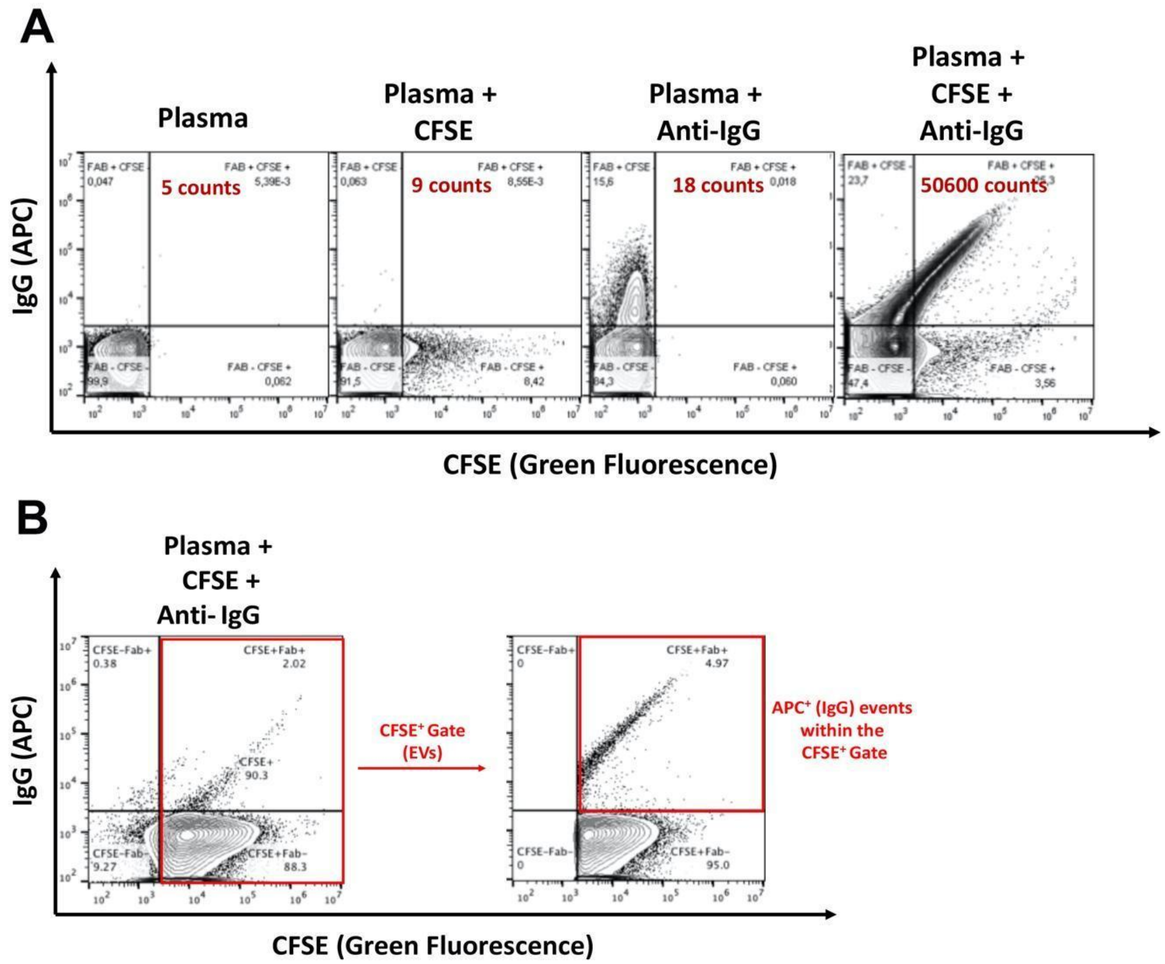

Supplementary Figure S1. Flow cytometry gating strategy. (**A**) Internal controls: representative plots of unstained plasma, plasma stained with CFSE, plasma stained with anti-IgG (FAB), and plasma stained with both CFSE and anti-IgG; the indicated counts correspond to the events within the upper right quadrant ($CFSE^+IgG^+$). (**B**) Characterization of EVs in plasma: representative plots of particles labeled with CFSE and anti-IgG (FAB) from plasma; the right panel indicates the IgG^+ (top right quadrant) and IgG^- (lower right quadrant) events within $CFSE^+$ particles.

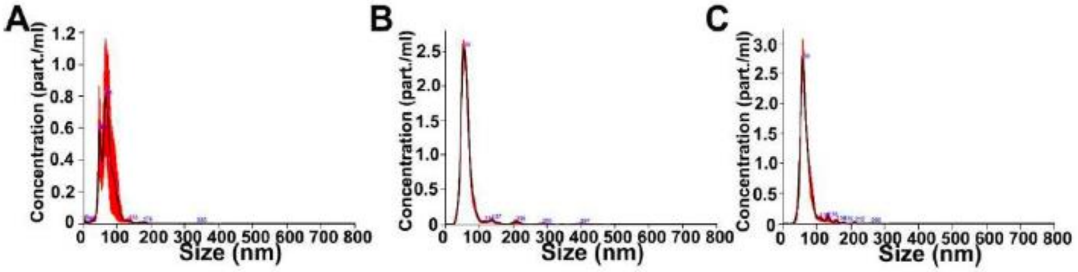

D

	Protein	Healthy	PDAC
Proteins commonly present in small EVs	Actin B	+	+
	Alix	-	-
	CD9	+	+
	HSPA8	+	+
	GAPDH	+	+
	Syntenin	+	+
Proteins commonly present in large	ACTN4	-	-
	MIC60	-	-
	RGAP1	-	-
Non-EV proteins	Bcl-2	-	-
	Calnexin	-	-
	GM130	-	-
	Nucleoporin	-	-

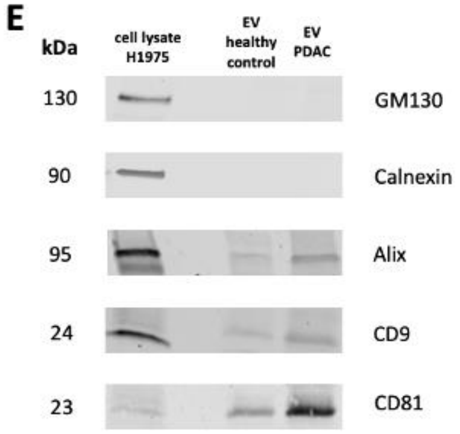

Supplementary Figure S2. Characterization of EVs. Representative size distribution histograms by Nanoparticle Tracking Analysis (NTA) of samples isolated from healthy controls (**A**) and from PDAC patients at diagnosis that did not respond (**B**) or that responded (**C**) to therapy. (**D**) Panel depicting the presence status of the indicated proteins in isolated EVs. (**E**) Representative Western blots of EV-markers CD9, CD81, Alix and non-EV markers GM130 and Calnexin for EV samples isolated from healthy control and PDAC plasma (H1975 cell lysate was used as a control).

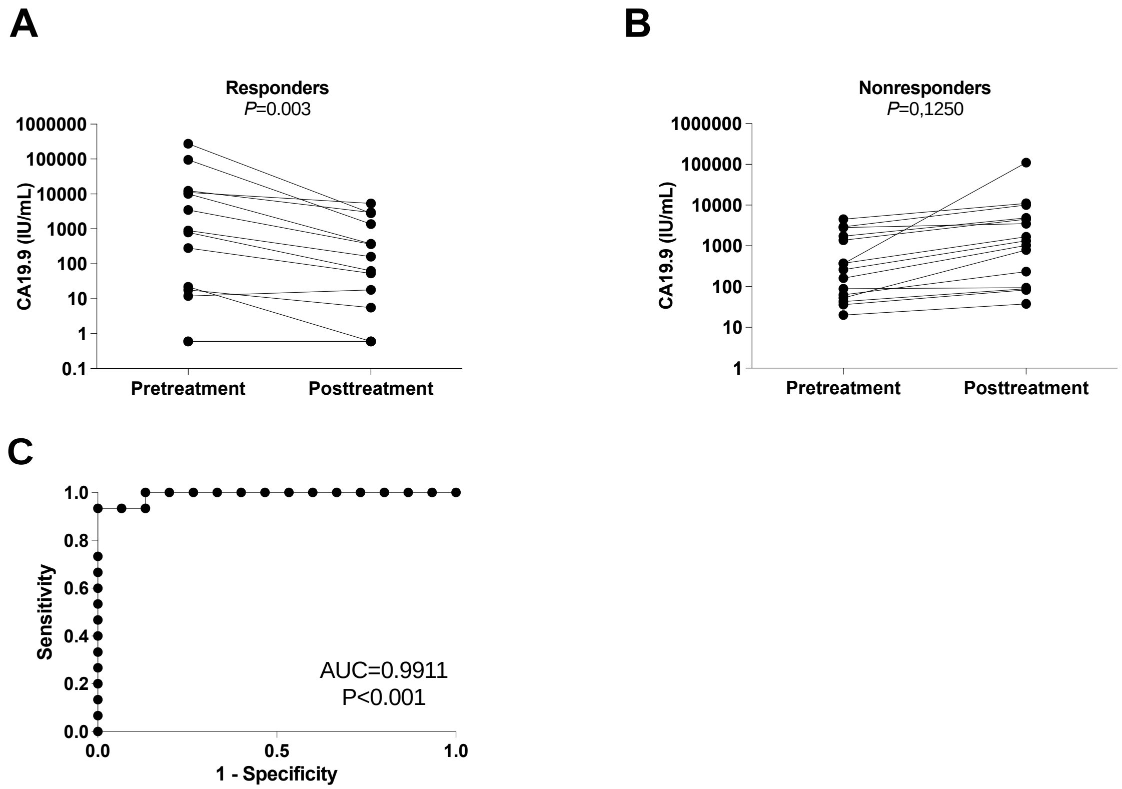

Supplementary Figure S3. CA19.9 levels in PDAC patients. Evolution of CA19.9 before and after treatment with chemotherapy in responders (**A**) and nonresponders (**B**) Depicted are the same patients and timepoints as in Figure 4 C and D. Wilcoxon test was used for statistical analysis. (**C**) ROC curve for the CA19.9 to discriminate response in patients with metastatic PDAC.

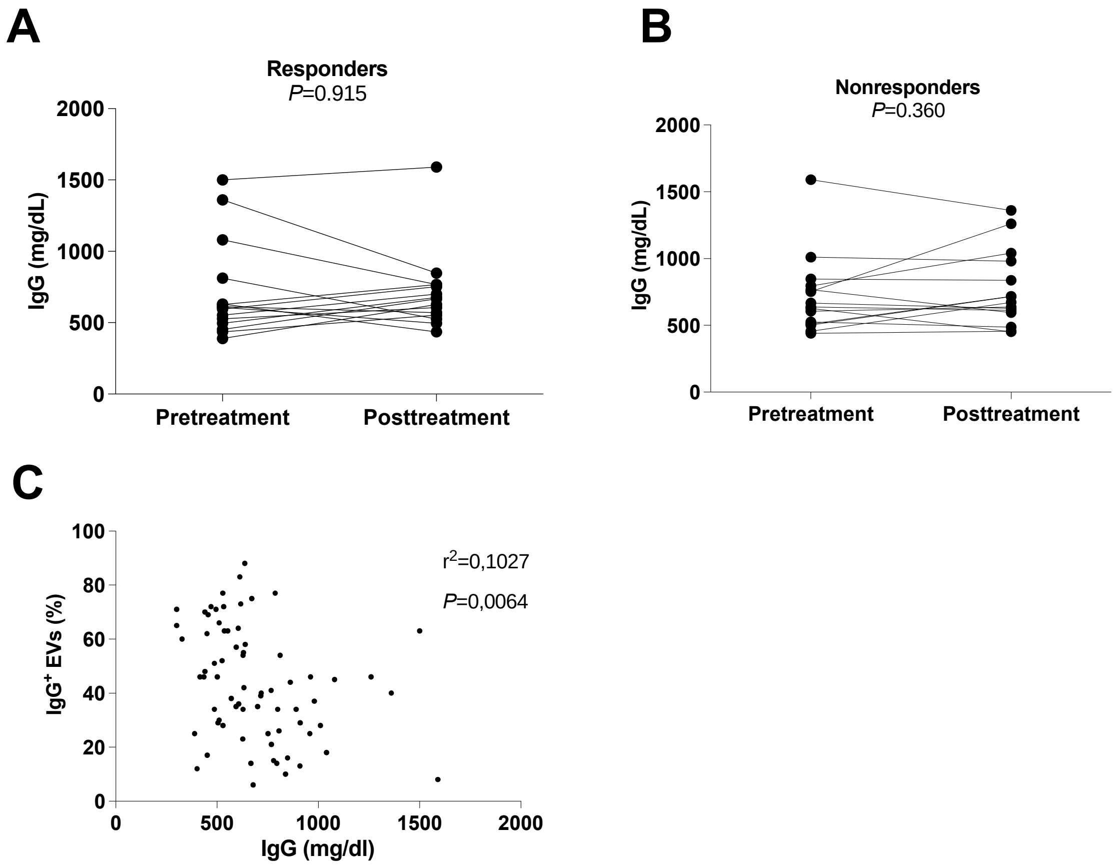

Supplementary Figure S4. Plasmatic IgG levels during treatment of PDAC patients. Comparison between plasmatic levels of IgG before and after treatment with chemotherapy in responders, by Wilcoxon test (**A**) and nonresponders, by t-test (**B**) Depicted are the same patients and timepoints as in Figure 4 C and D. There were no significant differences in both groups. (**C**) Correlation of the same time points of plasmatic IgG (mg/dL) vs percentage of IgG^+ EVs. Statistical analysis was performed by simple linear regression.

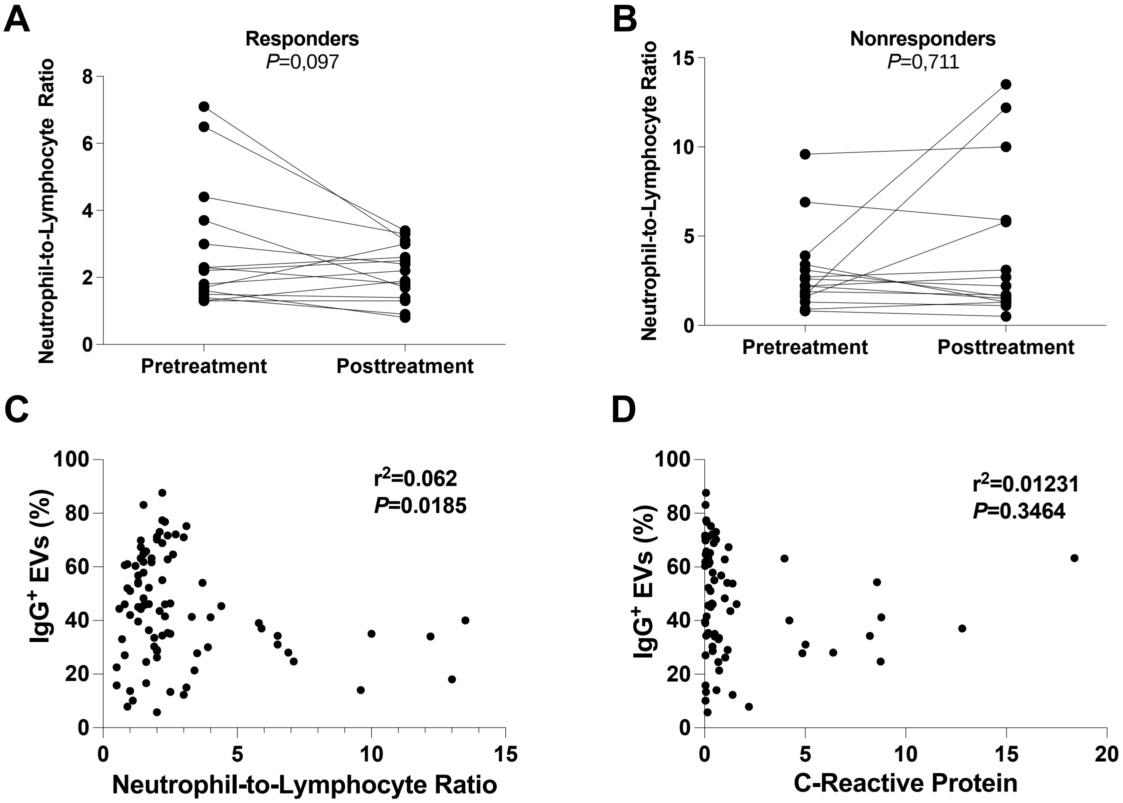

Supplementary Figure S5. Assessment of the inflammatory status of PDAC patients. Evolution of the neutrophil/lymphocyte ratio (NLR) between timepoints pre and post treatment with chemotherapy in responders, by t-test (**A**) and nonresponders, by Wilcoxon test (**B**). Depicted are the same patients and timepoints as in Figure 4 C and D. There were no significant differences in both groups. Correlation of the same time points of IgG^+ EVs vs NLR (**C**) and vs C-Reactive Protein (CRP) (**D**) Statistical analysis was performed by simple linear regression.

.

Supplementary Table S1 – List of primary and secondary antibodies used for Western blotting

**Primary Antibodies**			
**Antibody**	**Manufacturer**	**Catalog no**	**Dilution/concentration**
**CD81**	Santa Cruz Biotechnology	sc-166029	1:50
**CD9**	Cell Signaling	#13174	1:1000
**Alix**	Sigma Aldrich	SAB4200476-200UL	1.25 ug/mL
**Calnexin**	Abcam	ab22595	1:2000
**GM130**	Abcam	ab52649	1:1000
**Secondary Antibodies**			
**Antibody**	**Manufacturer**	**Catalog no**	**Dilution**
**Goat anti-rabbit IgG IRDye 800CW**	LI-COR Biosciences	926-32211	1:5000
**Goat anti-mouse IgG IRDye 800CW**	LI-COR Biosciences	926-32210	1:5000

Supplementary Table S2 – Cytometer configuration and laser power

Channel number	Short Channel Name	Full Channel Name	Optical Filter Name	Laser Wavelength	Laser Power	PMT Voltage
**Ch1**	405-SALS	Small Angle Light Scatter		405 nm	200 mW	400 V
**Ch2**	405-LALS	Large Angle Light Scatter		405 nm	200 mW	400 V
**Ch3**	405-Gm	Green Fluorescence	BP-525/50	405 nm	200 mW	500 V
**Ch4**	405-Org	Orange Fluorescence	LWP-590/35	405 nm	200 mW	500 V
**Ch5**	APC	Red Fluorescence	BP-676/36	638 nm	150 mW	550 V
**Ch6**	CFSE	Green Fluorescence	BP-525/50	488 nm	200 mW	525 V
**Ch7**	PE	Orange Fluorescence	BP-575/30	488 nm	200 mW	500 V
**Ch8**	488-Red	Red Fluorescence	BP-676/36	488 nm	200 mW	500 V

Ch9	488-DRed	Deep Red Fluorescence	LWP-740	488 nm	200 mW	500 V

Supplementary Table S3 – EV proteins regulated in patients with PDAC (vs. healthy controls)

Protein IDs	Gene names	Mean Healthy Controls	Mean PDAC	logFC	P.Value
P02675	FGB	30,80916	32,63287	1,823711	1,7E-15
P01591	IGJ	31,04684	29,11787	-1,92898	1,71E-12
Q16610;Q16610-4;Q16610-2;Q16610-3	ECM1	22,3285	24,74706	2,418563	5,83E-10
P01871;P01871-2	IGHM	32,80839	31,13976	-1,66863	3,11E-09
P02679	FGG	31,73688	32,79369	1,056811	5,31E-09
P07360	C8G	24,25857	25,95475	1,69618	1,28E-08
Q9Y2I7	PIKFYVE	8.610452	18,488365	9,877913	5,23E-08
P08603;P08603-2	CFH	25,70408	26,9523	1,24822	8,16E-08
P02751;P02751-8;P02751-3;P02751-15	FN1	29,12809	30,96719	1,839091	8,28E-08
O75636;O75636-2	FCN3	27,6463	30,25062	2,604322	1,07E-07
P12111-4;P12111-2;P12111;P12111-3;P12111-5	COL6A3	0.7664544	13,3798081	12,61335	1,14E-07
Q9C0K7-3;Q9C0K7-2;Q9C0K7	STRADB	6.21537	19,08732	12,87195	1,86E-07
P81605;P81605-2	DCD	15,62981	1.59704	-14,0328	2,21E-07
P00488;P16452-3;P16452;P16452-2	F13A1	23,53023	25,2402	1,709968	2,47E-07
A0A0B4J1V6	IGHV3-73	24,99557	23,20821	-1,78736	3,07E-07
P09871	C1S	22,88309	23,99478	1,111685	3,33E-07
P02679-2	FGG	13,1101	21,87546	8,765356	6,49E-07
O43866	CD5L	29,42484	27,78712	-1,63772	8,76E-07
Q08830	FGL1	13,01631	21,4914	8,475092	9,83E-07
P0DOY3		32,86217	31,9975	-0,86467	1,03E-06
P01709		27,85825	25,97132	-1,88692	1,71E-06
A0A0B4J1V0	IGHV3-15	25,76245	24,63909	-1,12337	2,45E-06
P00451;P00451-2	F8	18,60007	21,25979	2,659721	3,93E-06
P07358	C8B	23,55035	24,45246	0,902108	5,32E-06
P01011;P01011-2	SERPINA3	24,59445	25,81344	1,218999	1E-05
P01042	KNG1	22,39664	23,79217	1,395532	1,05E-05
O14791;O14791-2;O14791-3	APOL1	26,07611	24,37361	-1,7025	1,76E-05
P04275;P04275-2	VWF	27,3174	29,24253	1,925138	1,9E-05
P04196	HRG	24,01787	25,45884	1,440968	2,94E-05
P12109	COL6A1	5.053083	15,624026	10,57094	3,88E-05
P01780		29,81985	28,87501	-0,94484	6,6E-05
A0A087WW87;P01614	IGKV2-40	28,98482	28,20821	-0,77661	9,96E-05
P06312	IGKV4-1	27,12003	26,34662	-0,77341	0,00014
P04211		23,414648	9.843152	-13,5715	0,000152
Q08380	LGALS3BP	27,12358	25,96748	-1,1561	0,000168
P13671	C6	22,07315	23,13764	1,064495	0,000171

P10909-4;P10909;P10909-5;P10909-2;P10909-3	CLU	26,44996	27,21772	0,767758	0,000203
A0M8Q6	IGLC7	16,343175	4.559787	-11,7834	0,000234
P02792	FTL	23,44187	26,07389	2,632021	0,000249
Q4LDE5;Q4LDE5-4;Q4LDE5-3;Q4LDE5-2	SVEP1	4.153104	14,13788	9,984777	0,000271
Q9HCU4	CELSR2	7.209845	18,779373	11,56953	0,000376
Q14520-2;Q14520	HABP2	9.420802	18,345494	8,924692	0,000449
Q9Y6R7	FCGBP	22,3186	20,91387	-1,40473	0,000479
Q9NZT1	CALML5	15,381387	4.785034	-10,5964	0,000542
Q08554-2;Q08554	DSC1	12,67823	3.226449	-9,45178	0,000723
Q96IY4;Q96IY4-2	CPB2	9.124999	18,288738	9,163739	0,000772
P01009;P01009-2;P01009-3;P20848	SERPINA1	27,45175	28,67493	1,223177	0,000823
A0A0C4DH68	IGKV2-24	27,98671	27,01923	-0,96748	0,00084
P02743	APCS	26,49926	27,12377	0,624511	0,000918
O43166	SIPA1L1	3,611292	0	-3,61129	0,001353
P01859	IGHG2	28,50515	29,53058	1,025437	0,001748
Q01469	FABP5	13,333104	3.547731	-9,78537	0,001785
Q562R1	ACTBL2	4,454414	0	-4,45441	0,001804
A0A0A0MRZ9	IGLV5-52	4,580166	0	-4,58017	0,001869
P01860	IGHG3	28,57457	29,43901	0,86444	0,002166
P02763	ORM1	23,88044	25,18618	1,30574	0,002263
P29622	SERPINA4	15,58681	20,29616	4,709354	0,002959
O00189	AP4M1	8.074653	16,858605	8,783952	0,003489
P07357	C8A	24,73622	25,25004	0,513816	0,004064
P01594;P01593		23,88627	24,80415	0,91788	0,004506
Q15582	TGFBI	5.345572	13,679386	8,333814	0,005173
P98160	HSPG2	8.859966	15,662051	6,802085	0,005736
A2NJV5;A0A075B6S2;A0A0A0MRZ7	IGKV A18;IGKV2D-29;IGKV2D-26	6,2640694	0,5281823	-5,73589	0,006043
P00748	F12	21,33945	22,1381	0,798658	0,006085
P22792	CPN2	22,25886	22,8205	0,561642	0,006173
P11021	HSPA5	3.720965	11,713255	7,992289	0,006826
P01876	IGHA1	31,13867	30,24995	-0,88872	0,006911
Q92496-2;Q92496;Q92496-3	CFHR4	2.42436	11,03821	8,613846	0,007766
A0A075B6I1	IGLV4-60	13,87167	5.185394	-8,68628	0,010051
P01703		17,37782	23,23569	5,857872	0,010649
P18428	LBP	11,47416	18,31519	6,841032	0,011323
Q8NI99	ANGPTL6	11,526017	4.144387	-7,38163	0,011906
A0A0B4J1U7	IGHV6-1	22,59996	26,62302	4,023052	0,012004
Q9BXR6	CFHR5	5.800607	13,338935	7,538328	0,014154
P02751-10;P02751-13	FN1	9.087818	16,69571	7,607892	0,01441
P02790	HPX	25,28332	26,11439	0,831076	0,014443
P21980-2;P21980;P21980-3	TGM2	4,2720915	0,4457934	-3,8263	0,014565
P02746	C1QB	27,56949	28,01983	0,450336	0,019773
P02760	AMBP	24,33717	24,93431	0,597142	0,020355
P01701		27,48918	26,60877	-0,88041	0,021183
P01766		24,45966	17,99343	-6,46623	0,023374
P01019	AGT	23,4802	22,67968	-0,80053	0,023513
A0A0G2JS06		25,3019	19,47542	-5,82649	0,024552
P01704		19,9328	25,27153	5,338733	0,024827
Q5T749	KPRP	4,4687764	0,5660902	-3,90269	0,024872
O00187;O00187-2	MASP2	19,93343	20,97891	1,04548	0,026433
Q03591	CFHR1	23,1884	23,76836	0,579961	0,02717
P11217-2;P11217;P06737-2;P11216;P06737	PYGM	4,6574862	0,6082011	-4,04929	0,027593

P07225	PROS1	25,57112	25,99843	0,427312	0,029207
Q12805-5;Q12805-2;Q12805-4;Q12805-3;Q12805	EFEMP1	2,296374	8,973493	6,67712	0,029245
A0A075B6H9	IGLV4-69	25,56016	20,4878	-5,07236	0,029928
P01715		19,30839	11,32053	-7,98786	0,031146
Q02413;Q02413-2	DSG1	3,6552677	0,5459463	-3,10932	0,036858
Q66K66	TMEM198	20,92864	23,50407	2,575437	0,038233
P08473	MME	15,310028	8.574814	-6,73521	0,038239
P68133;P68032;P63267;P62736;P63267-2	ACTA1;ACTC1;ACTG2;ACTA2	7.430236	13,875415	6,44518	0,040955
A0A0C4DH24	IGKV6-21	24,16357	18,66946	-5,4941	0,041297
P01817		12,696209	5.607219	-7,08899	0,042827
P15144	ANPEP	20,31834	14,37981	-5,93853	0,045148
P62987;P62979;P0CG47;P0CG48	UBA52;RPS27A;UBB;UBC	19,56519	12,92859	-6,63661	0,046162
A0A075B6S6	IGKV2D-30	12,474057	5.802547	-6,67151	0,048532
P08519;Q16609	LPA	5.945479	12,500058	6,554579	0,049001

Supplementary Table S4 – EV proteins regulated in nonresponders PDAC patients (vs. responders)

Protein IDs	Gene names	Mean Non Responders	Mean Responders	logFC	P.Value
A0A0C4DH72;A0A0C4DH73;P01611	IGKV1-6	15,060969	3.574371	-11,4866	0,000489
P04432;P01597		14,692537	3.558026	-11,1345	0,000562
P01817		10,90292	0.00000	-10,9029	0,000293
A0A0C4DH68	IGKV2-24	26,58469	27,47934	0,894657	0,000201
P02746	C1QB	27,77124	28,28304	0,511799	0,001447
P01714		25,10489	26,84546	1,740563	0,00226
P15814;P15814-2	IGLL1	8,060513	0	-8,06051	0,002832
Q08554-2;Q08554	DSC1	0	6,642689	6,642689	0,004575
A0A075B6S5;A0A0C4DH69	IGKV1-27	15,14843	5.662179	-9,48625	0,004863
P62987;P62979;P0CG47;P0CG48	UBA52;RPS27A;UBB;UBC	8.622959	17,487488	8,864529	0,007196
P15144	ANPEP	10,49458	18,49358	7,998998	0,009811
A0A0B4J1V0	IGHV3-15	24,3923	24,90039	0,508095	0,009961
Q9NZP8	C1RL	9,544	2,343031	-7,20097	0,010317
A0A0A0MT36	IGKV6D-21	10,839119	2.553621	-8,2855	0,013266
P07357	C8A	25,08541	25,42435	0,338941	0,013604

Q13103	SPP2	9,603383	2,31008	-7,2933	0,014323
Q9BXR6	CFHR5	16,619122	9.865795	-6,75333	0,017651
Q8TCG1-2;Q8TCG1	KIAA1524	8,173923	1,876418	-6,2975	0,020795
P08637;O75015	FCGR3A	11,79168	18,71712	6,925439	0,021632
Q9BWP8-8;Q9BWP8-7;Q9BWP8-6;Q9BWP8-5	COLEC11	12,04857	4.62706	-7,42151	0,023107
P01857	IGHG1	32,56523	31,99845	-0,56677	0,02629
P03952;P20718	KLKB1	23,64847	23,94836	0,299895	0,026916
P13164;Q01629;Q01628	IFITM1;IFITM2;IFITM3	0	5,501942	5,501942	0,027497
P05106-2;P05106-3;P05106	ITGB3	0	3,959515	3,959515	0,027508
P02745	C1QA	27,34568	27,7409	0,395215	0,02757
A0A0C4DH43;P01814		23,72722	17,35704	-6,37018	0,028112
A0A0A0MS15	IGHV3-49	23,7816	22,93867	-0,84293	0,028814
P07225	PROS1	25,81825	26,18921	0,370953	0,028895
P04278-4;P04278-2;P04278-3;P04278;P04278-5	SHBG	13,2751	6.543573	-6,73153	0,029683
P08670;P17661;Q16352;P07197-2	VIM	0	4,58408	4,58408	0,029827
P02747	C1QC	28,01889	28,39234	0,373451	0,030901
P23083		25,56806	26,25761	0,689546	0,031442
P28074-3;P28074	PSMB5	8,442728	2,336585	-6,10614	0,03599
P04430		21,55277	16,68508	-4,8677	0,036212
A0A087WSX0	IGLV5-45	5,619149	0	-5,61915	0,037874
P01699		4,512501	0	-4,5125	0,037909
P27169	PON1	23,04364	23,63591	0,592268	0,040998
A0A0C4DH29	IGHV1-3	21,04053	15,33251	-5,70801	0,042131
A0A075B6I0	IGLV8-61	25,2758	19,73274	-5,54307	0,04525
A0A075B6Q5	IGHV3-64	18,17808	11,41315	-6,76493	0,047306
Q01469	FABP5	1,009046	6,235751	5,226705	0,04767
P01861	IGHG4	27,21168	26,56055	-0,65113	0,048619
P00739;P00739-2	HPR	26,29785	25,7406	-0,55725	0,049185
